# Zafirlukast ameliorates Docetaxel-induced activation of NOD-like receptor protein 3 (NLRP3) inflammasome, mediated by sirtuin1 (SIRT1) in hepatocytes

**DOI:** 10.1080/21655979.2021.2005895

**Published:** 2021-11-30

**Authors:** Ziyi Guo, Xunjin Zeng, Yu Zheng

**Affiliations:** Intervention Centre, the First Affiliated Hospital of Jinzhou Medical University, Jinzhou City, Liaoning Province, China

**Keywords:** Zafirlukast, Docetaxel, hepatocytes, LDH, SIRT1

## Abstract

Docetaxel-associated liver injury has become a serious public health problem, resulting in therapy discontinuation, liver failure, and death. Zafirlukast is a typical leukotriene receptor antagonist used for prophylaxis and chronic treatment of asthma. In this study, we investigate whether treatment with Zafirlukast could alleviate Docetaxel-induced cytotoxicity in hepatocytes. Our results indicate that Zafirlukast mitigated Docetaxel-induced toxicity in LO-2 hepatocytes. Firstly, Zafirlukast reduced the production of 8-hydroxy-2p-deoxyguanosine (8-OHdG) and increased the levels of reduced glutathione (GSH) against Docetaxel. Secondly, Zafirlukast elevated the levels of mitochondrial membrane potential (ΔΨm) and adenosine triphosphate (ATP). Thirdly, Zafirlukast prevented Docetaxel-induced release of lactate dehydrogenase (LDH) and increased cell viability of LO-2 hepatocytes against Docetaxel. We also found that Zafirlukast ameliorated Docetaxel-induced apoptosis by reducing Caspase-3 and Caspase-9 activity. Mechanistically, our results demonstrate that Zafirlukast inhibited the activation of NOD-like receptor protein 3 (NLRP3), mediated by SIRT1. Based on these findings, we conclude that the administration of Zafirlukast might have a protective effect against Docetaxel-induced cytotoxicity in hepatocytes.

## Introduction

Drug-induced liver injury (DILI) is a common side effect observed clinically that can progress to chronic liver injury, liver fibrosis, and even liver failure or death [1,2]. DILI is defined as a disease induced by abnormal liver function or hypersensitivity reactions triggered by drugs or their metabolites [3]. It is reported that hepatotoxicity can be induced by approximately 1100 types of drugs [4]. DILI is mainly clinically characterized by fatigue, loss of appetite, yellow urine, and nausea, all of which may adversely impact the lives of patients [5]. The diagnosis and prevention of DILI have become global public health problems due to the high morbidity and a large number of patients. Russmann [6] divided the progression of DILI into 3 steps: the immune-inflammatory response directly induced by drugs or their metabolites, changes of the mitochondrial membrane permeability, and decreased adenosine triphosphate synthesis. Ultimately, necrosis and apoptosis of hepatocytes are induced. Direct damage or oxidative stress induced by drugs or their metabolites are the main mechanisms of mitochondrial damage in the progression of DILI [7]. Firstly, the mitochondrial respiratory chain is inhibited by drugs or their metabolites to block the synthesis of ATP and decrease the energy supply. Subsequently, the anti-oxidative system in hepatocytes is disrupted by drug-induced excessive release of reactive oxygen species (ROS). In addition, the accumulation of large amounts of fatty acids is induced by the declined β-oxidation of fatty acids, which further triggers the hepatocytic steatosis, damages the mitochondrial DNA, affects the expression levels of mitochondrial genes or proteins, and inhibits the regeneration and repair of mitochondria. As a consequence, the permeability of the mitochondrial membrane is increased, likewise, the calcium influx and the oxidative reaction in the mitochondria is activated to induce the mitochondrial dysfunction [8–10]. Apart from mitochondrial dysfunction, severe inflammation in hepatocytes is another pathological stimulator for DILI. It is reported that the activation of the NLRP3 inflammasome, an important inflammatory pathway, is induced in multiple types of DILI, such as D-galactosamine [11], acetaminophen [12], and cisplatin [13]. SIRT1, an inhibitory transcriptional factor for the NLRP3 inflammasome, is reported to act as a protective mediator for DILI [14]. Docetaxel, a chemotherapeutic drug widely applied for the treatment of malignant tumors, is reported to induce severe liver injury during clinical treatments [15,16]. It is of great significance to develop effective therapies for the DILI during the Docetaxel treatments.

Zafirlukast is a highly selective leukotriene receptor antagonist applied for the treatment of asthma. It effectively prevents the increase in vascular permeability, airway edema, and bronchial smooth muscle contraction. It also inhibits the infiltration of eosinophils and lymphocytes and reduces the peroxides secreted following the alveolar macrophage stimulation [17]. Recently, significant anti-inflammatory [18], and inhibitory effects against oxidative stress [18] properties of Zafirlukast have been claimed. The present study will explore the potential protective effects of Zafirlukast on Docetaxel-induced injury in hepatocytes and provide the fundamental basis for its possible application of Zafirlukast for the treatment of Docetaxel-induced liver injury in clinics.

## Materials and methods

### Cell culture, treatment, and transduction

LO-2 hepatocytes were obtained from BNCC (Beijing, China), and cultured in the DMEM medium containing 100 mg/ml streptomycin, 10% FBS, and 100 IU/ml penicillin. To obtain the SIRT1-knockdown hepatocytes, LO-2 hepatocytes were transduced with lentiviral SIRT1 shRNA designed and synthesized by Genscript (Nanjing, China). The efficacy of transduction was confirmed by the Western blotting assay.

### Determination of reduced GSH

LO-2 hepatocytes were collected and the supernatant was obtained for the detection of the concentration of reduced GSH according to the methods described by Beutler [19].

### Dichlorodihydrofluorescein diacetate (DCFH-DA) staining for ROS

LO-2 hepatocytes were seeded on a 96-well plate. After treatment, cells were washed with PBS and loaded with 10 μM DCFH-DA. After incubation for 30 min in the darkness, fluorescent intensity was measured at 488/525 nm wavelength using a microplate reader (Molecular Devices, California, USA).

### Rhodamine 123 (RH123) staining

Approximately 5 × 10^5^ cells were collected and resuspended in culture medium, followed by adding RH123 staining reagents (Shanghai Hengyuan Bio, Shanghai, China). After incubating at 37°C for 30 min, the staining reagents were removed and cells were washed using PBS buffer. Lastly, the fluorescence microscope (Zeiss, Aalen, Germany) was used for the observation [20].

### Measurement of intracellular ATP

A commercial ATP detection kit (Roche, Basel, Switzerland) was utilized for measuring the release of intracellular ATP in hepatocytes. In brief, hepatocytes were implanted into a 6-well plate and the lysis buffer was added. Then, samples were centrifugated at 12,000 g under the temperature of 4°C, followed by collecting the supernatant for subsequent detection. The ATP working solution was added to the detecting well to be incubated at room temperature for 5 min, followed by adding 20 μL samples or standards and mixing [21]. Lastly, the luminometer (Beckman, California, USA) was used to measure the value of the relative light unit (RLU) and the concentration of intracellular ATP was calculated according to the standard curve.

### LDH release

The release of LDH in hepatocytes was determined using the CytoTox-ONE™ kit (Promega, Wisconsin, USA). Briefly, the supernatant of hepatocytes was collected and transferred to a 96-well plate, followed by adding the CytoTox-ONE™ reagent for 10 min incubation. Then, the stop solution was introduced, followed by measuring the absorbance at 560/590 nm using the microplate reader (BMG LABTECH, Offenburg, Germany) [22].

### The activity of caspase-3 and caspase-9

The commercial kits (Solarbio, Beijing, China) were used for the detection of the activity of Caspase-3 and Caspase-9. In brief, 7 concentrations of standards were obtained by diluting the 10 mM standard parent solution, then added into a 96-well plate pre-coded with substrates and incubation for 2 hours. Then, the absorbance at 405 nm was measured using the microplate reader (BMG LABTECH, Offenburg, Germany) to establish the standard curve. Hepatocytes were treated with lysis buffer, followed by the quantification of total proteins using the method of Bradford. For each sample, approximately 30 μg of proteins were added to the 96-well plates, followed by incubation at 37°C for 2 hours. Lastly, the absorbance at 405 nm was measured using the microplate reader (BMG LABTECH, Offenburg, Germany), and the activity of Caspase-3 and Caspase-9 was calculated according to the standard curve [23].

### Real-time PCR

The total RNAs were extracted from treated LO-2 hepatocytes with the TRIzol reagent (Sigma-Aldrich, California, USA), followed by transcribing 2 µg RNA into cDNA for each sample using the PrimeScript RT Reagent Kit (Takara, Tokyo, Japan). Then, the 7500 Real-Time PCR System (ABI, California, USA) using the SYBR-Green dye (ABI, California, USA) was utilized to conduct the PCR reaction, followed by determining the expression of genes with the 2^−ΔΔCt^ method.

### Western blot analysis

Treated hepatocytes were lysed to obtain the total proteins and the bicinchoninic acid (BCA) kit (Abcam, Cambridge, UK) was used for the quantification of extracted proteins. After loading proteins onto 12% SDS-PAGE, proteins were separated for 2 hours and then moved to PVDF membrane (Abcam, Cambridge, UK). The TBST buffer supplemented with the primary antibodies against NLRP3 (1:1000, GeneTex, Texas, USA), SIRT1 (1:1000, GeneTex, Texas, USA), and β-actin (1:1000, GeneTex, Texas, USA), followed by incubating with the secondary antibody (1:2000, GeneTex, Texas, USA) for 90 min. Finally, the membrane was exposed using the ECL solution, followed by quantifying the expression of proteins utilizing the Image J software.

### Enzyme-linked immunosorbent assay (ELISA)

The levels of 8-OHdG, interleukin (IL)-18, and IL-1β were measured using the ELISA assay (R&D Systems, Minnesota, USA). In brief, the supernatant of hepatocytes, as well as different concentrations of standards, were collected and seeded on a 96-well plate, followed by incubation at room temperature for 90 min. Then, the conjugate solution was introduced and samples were incubated for 90 min, followed by introducing the TMB solution for 15 min. Finally, the reaction was terminated by adding the stop solution and the absorbance at 450 nm was measured by a microplate reader (BMG LABTECH, Offenburg, Germany) [24].

### Statistical analysis

The GraphPad software was utilized to analyze data obtained in the present study, which were presented as mean ± standard errors (S.E.). The difference between the 2 groups was analyzed using the Student’s t-test and the ANOVA method was used to analyze the difference among more than 2 groups. P < 0.05 was taken as a significant difference.

## Results

In order to investigate the protective benefits of Zafirlukast in DILI, we employed Docetaxel-challenged LO-2 hepatocytes to mimic an *in vitro* DILI model. We examined the effects of Zafirlukast on Docetaxel-induced oxidative stress, mitochondrial dysfunction, apoptosis, and activation of the NLRP3 inflammasome. Additionally, to uncover the underlying mechanism, we investigated the involvement of SIRT1 in Docetaxel-challenged hepatocytes.

### Zafirlukast ameliorated Docetaxel-induced oxidative stress in LO-2 hepatocytes

**Figure 1.**
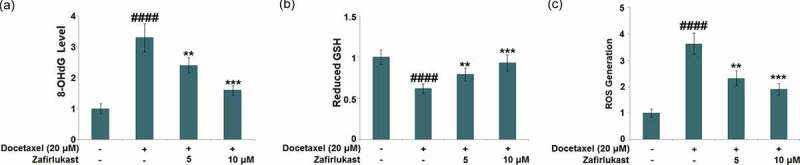
Zafirlukast ameliorated Docetaxel-induced oxidative stress in LO-2 hepatocytes. Cells were stimulated with Docetaxel (20 μM) in the presence or absence of Zafirlukast (5, 10 μM) for 24 hours. (a). The levels of 8-OHdG; (b). The levels of reduced GSH; (c). The levels of ROS generation (####, P < 0.0001 vs. control group; **, ***, P < 0.01, 0.005 vs. Docetaxel group)

Oxidative stress is an important inducer for the mitochondrial dysfunction in the progression of DILI. LO-2 hepatocytes were incubated with Docetaxel (20 μM) with or without Zafirlukast (5, 10 μM) for 24 hours, followed by evaluating the state of oxidative stress in hepatocytes [25,26]. We found that the level of 8-OHdG (Figure 1(a)) which was significantly increased in the Docetaxel group, which was greatly repressed by 5 and 10 μM Zafirlukast. Additionally, the decline in the level of reduced GSH (Figure 1(b)) in the Docetaxel group was dramatically reversed by 5 and 10 μM Zafirlukast. Furthermore, results in Figure 1(c) show that exposure to Docetaxel significantly increased the level of ROS generation, which was reduced by Zafirlukast. These results collectively reveal that oxidative stress in LO-2 hepatocytes induced by Docetaxel was alleviated by Zafirlukast.

### Zafirlukast mitigated Docetaxel-induced mitochondrial dysfunction in LO-2 hepatocytes

**Figure 2.**
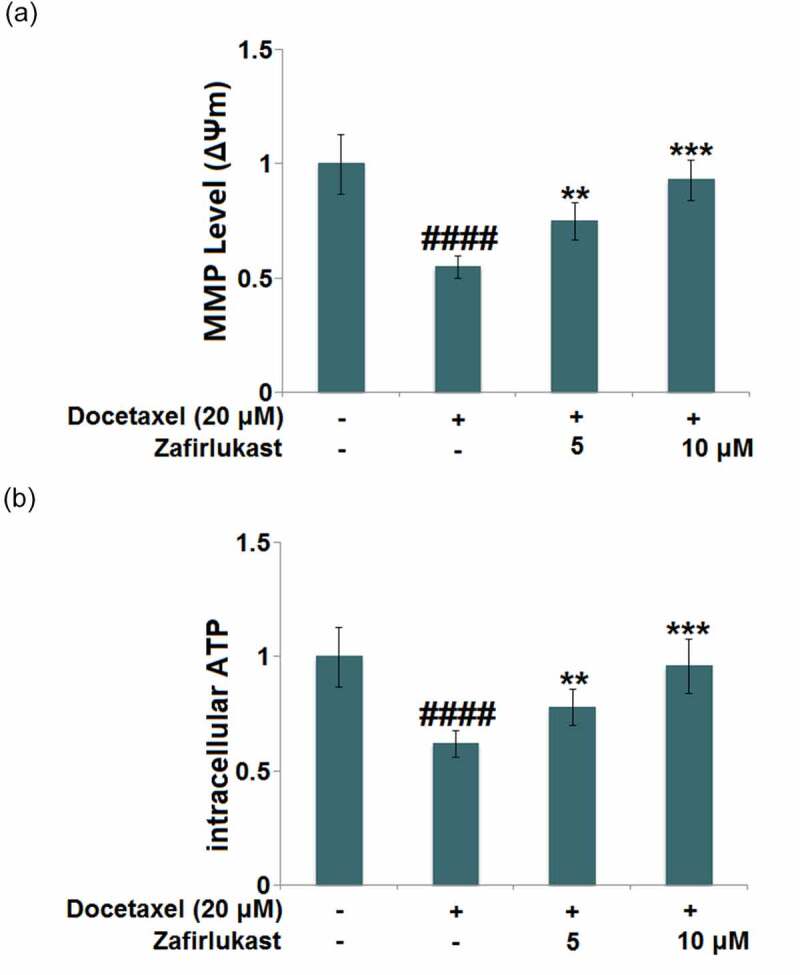
Zafirlukast mitigated Docetaxel-induced mitochondrial dysfunction in LO-2 hepatocytes. (a). Mitochondrial membrane potential (ΔΨm) was measured using RH123 staining; (b). The levels of intracellular ATP (####, P < 0.0001 vs. control group; **, ***, P < 0.01, 0.005 vs. Docetaxel group)

Mitochondrial dysfunction is mainly characterized by reduced mitochondrial membrane potential and declined ATP supply. After the stimulation with Docetaxel, the mitochondrial membrane potential (Figure 2(a)) was significantly reduced but was then greatly elevated by 5 and 10 μM Zafirlukast. In addition, the declined level of intracellular ATP (Figure 2(b)) in Docetaxel-treated hepatocytes was significantly promoted by 5 and 10 μM Zafirlukast, indicating the massive repair to the mitochondrial dysfunction in Docetaxel treated LO-2 hepatocytes was dramatically repaired by Zafirlukast.

### Zafirlukast attenuated the cytotoxicity of Docetaxel in LO-2 hepatocytes

**Figure 3.**
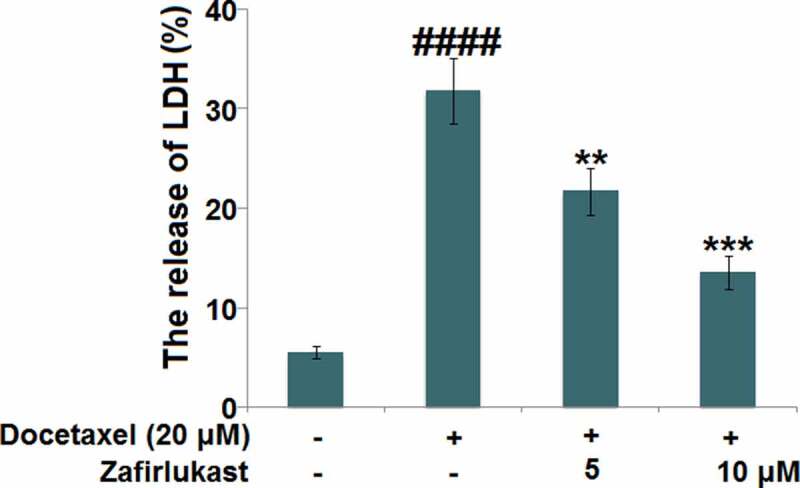
Zafirlukast attenuated the cytotoxicity of Docetaxel in LO-2 hepatocytes. The release of LDH (####, P < 0.0001 vs. control group; **, ***, P < 0.01, 0.005 vs. Docetaxel group)

We further investigated the protective effects of Zafirlukast on Docetaxel-induced cytotoxicity in hepatocytes. We found that the release of LDH (Figure 3) was significantly elevated from 5.5% to 31.8% by the treatment of Docetaxel, then greatly repressed to 21.7% and 13.6% by 5 and 10 μM Zafirlukast, respectively, indicating an effective protective property of Zafirlukast against Docetaxel-induced cytotoxicity in hepatocytes.

### Zafirlukast prevented Docetaxel-induced apoptosis in LO-2 hepatocytes

**Figure 4.**
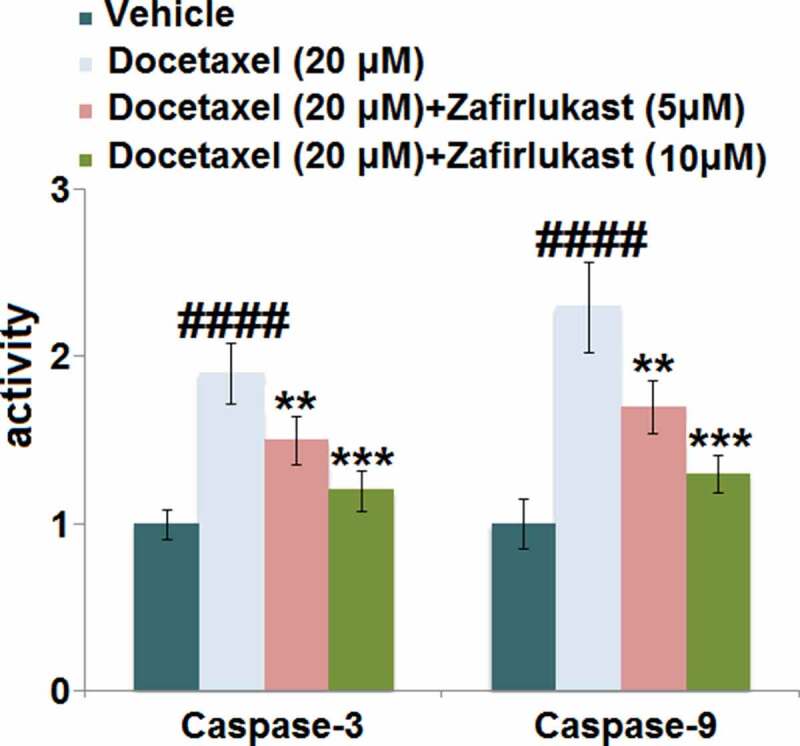
Zafirlukast prevented Docetaxel-induced apoptosis in LO-2 hepatocytes. The activity of Caspase-3 and Caspase-9 (####, P < 0.0001 vs. control group; **, ***, P < 0.01, 0.005 vs. Docetaxel group) was measured using commercial kits

Caspases are important effector molecules for the development of cell apoptosis. Our results indicate that in LO-2 hepatocytes, the activity of both caspase-3 and caspase-9 (Figure 4) was pronouncedly elevated by Docetaxel but greatly inhibited by 5 and 10 μM Zafirlukast, indicating that the apoptosis in Docetaxel-treated hepatocytes was antagonized by Zafirlukast.

### Zafirlukast prevented Docetaxel-induced activation of the NLRP3 inflammasome in LO-2 hepatocytes

**Figure 5.**
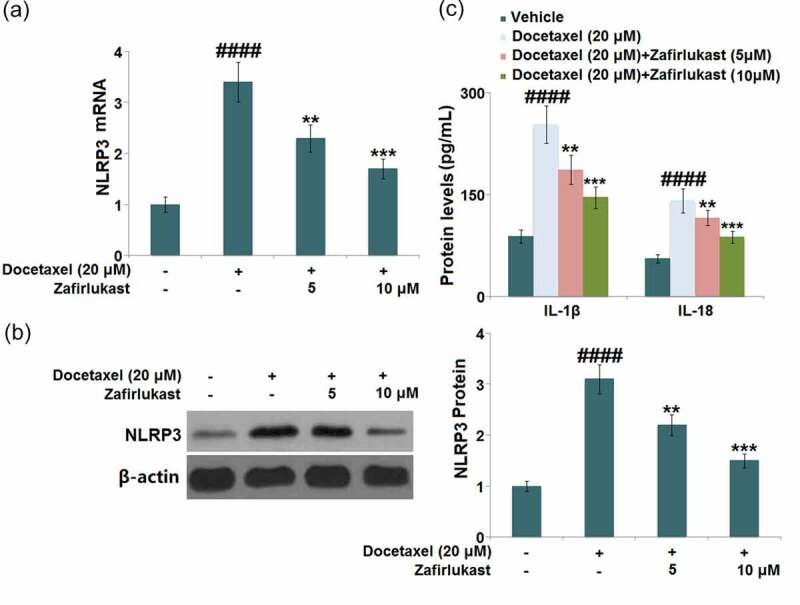
Zafirlukast prevented Docetaxel-induced activation of NLRP3 inflammasome in LO-2 hepatocytes. (a). mRNA of NLRP3; (b). Protein levels of NLRP3; (c). The levels of IL-1β and IL-18 (####, P < 0.0001 vs. control group; **, ***, P < 0.01, 0.005 vs. Docetaxel group)

NLRP3 inflammasome-mediated inflammation is reported to be a key inducer for DILI. The promoted expression level of NLRP3 (Figure 5(a-b) observed in Docetaxel-treated hepatocytes was found to be greatly suppressed by 5 and 10 μM Zafirlukast. The release of IL-1β was dramatically increased from 88.3 pg/mL to 253.5 pg/mL in Docetaxel-stimulated hepatocytes, then declined to 186.6 pg/mL and 145.9 pg/mL by 5 and 10 μM Zafirlukast, respectively. In addition, the release of IL-18 in the control, Docetaxel, 5 μM Zafirlukast, and 10 μM Zafirlukast groups was 56.2, 141.3, 115.7, and 87.3 pg/mL, respectively (Figure 5(c). We suspect that the NLRP3 inflammasome-mediated inflammation in Docetaxel-treated hepatocytes was significantly ameliorated by Zafirlukast.

### Zafirlukast restored Docetaxel-induced reduction of SIRT1 in LO-2 hepatocytes

**Figure 6.**
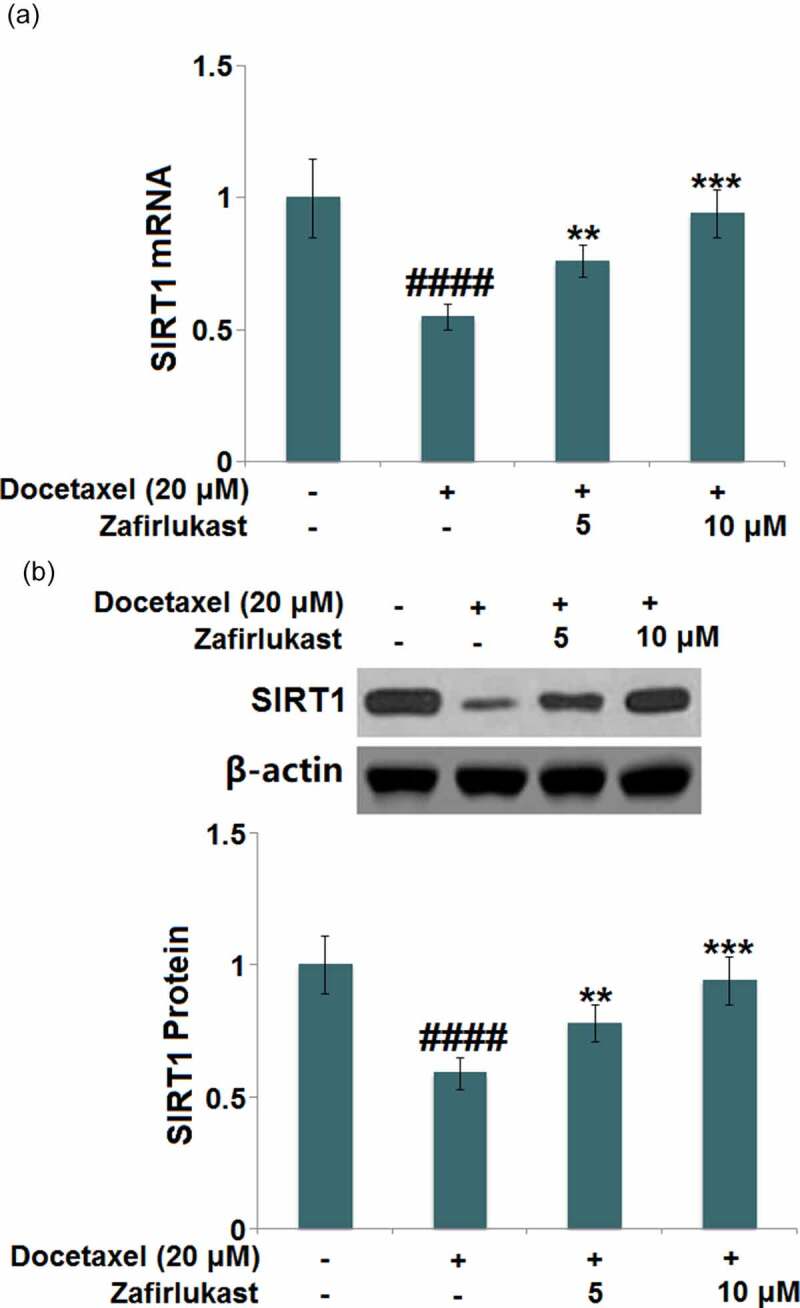
Zafirlukast restored Docetaxel-induced reduction of SIRT1 in LO-2 hepatocytes. (a). mRNA of SIRT1; (b). Protein levels of SIRT1 (####, P < 0.0001 vs. control group; **, ***, P < 0.01, 0.005 vs. Docetaxel group)

SIRT1 is reported to be a critical anti-inflammatory transcriptional factor, which exerts inhibitory effects on the NLRP3 inflammasome pathway. We found that SIRT1 (Figure 6(a-b) was dramatically downregulated in Docetaxel-treated hepatocytes but greatly upregulated by 5 and 10 μM Zafirlukast, indicating an effective activation impact of Zafirlukast on the transcriptional factor SIRT1.

### Silencing of SIRT1 abolished the protective property of Zafirlukast against Docetaxel-induced damages in LO-2 hepatocytes

**Figure 7.**
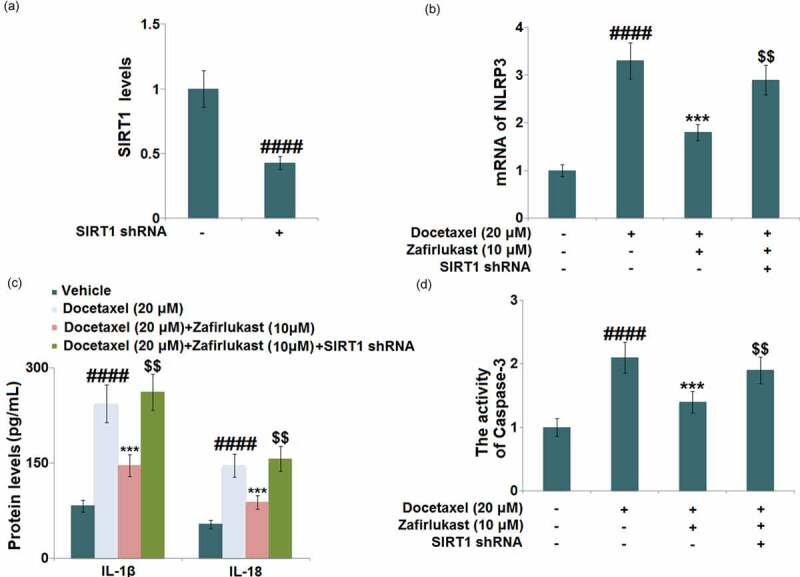
Silencing of SIRT1 abolished the protective effects of Zafirlukast against Docetaxel-induced damages in LO-2 hepatocytes. Cells were transduced with lentiviral SIRT1 shRNA, followed by stimulation with Docetaxel (20 μM) in the presence or absence of Zafirlukast (10 μM) for 24 hours. (a). Real time PCR revealed successful knockdown of SIRT1; (b). mRNA of NLRP3; (c). The levels of IL-1β and IL-18; (d). The activity of Caspase-3 (####, P < 0.0001 vs. control group; ***, P < 0.005 vs. Docetaxel group; $$, P < 0.01 vs. Docetaxel+ Zafirlukast group)

To further identify the involvement of SIRT1 in the regulatory mechanism of Zafirlukast, hepatocytes were transfected with lentiviral SIRT1 shRNA, followed by incubation with Docetaxel (20 μM) with or without Zafirlukast (10 μM) for 24 hours. Firstly, the efficacy of SIRT1 knockdown was identified by real time PCR (Figure 7(a)). The upregulated NLRP3 (Figure 7(b)) in Docetaxel-treated hepatocytes was inhibited by Zafirlukast, which was greatly abrogated by the knockdown of SIRT1. Additionally, the release of IL-1β (Figure 7(c)) in the control, Docetaxel, Zafirlukast, and Zafirlukast + SIRT1 shRNA groups was 82.6, 243.5, 146.2, and 262.1 pg/mL, respectively. The production of IL-18 was promoted from 53.7 pg/mL to 146.6 pg/mL in Docetaxel-stimulated hepatocytes, which was then greatly repressed to 88.3 pg/mL by 10 μM Zafirlukast. After the transfection with SIRT1 shRNA, the release of IL-18 was dramatically reversed to 156.8 pg/mL. Lastly, we found that the inhibitory effect of Zafirlukast on the activity of caspase-3 was significantly abolished by the knockdown of SIRT1 (Figure 7(d)). These data collectively indicate that the protective effects of Zafirlukast against Docetaxel- induced damages in LO-2 hepatocytes were greatly abolished by the silencing of SIRT1.

## Discussion

Mitochondria are important organelles that function to synthesize ATP. They are also reported to be involved in the regulation of ROS, a series of cellular signals, cell apoptosis, and autophagy cascade signal initiation, and other important biological processes. It is widely accepted that mitochondria are not only the ‘stabilizers’ to maintain the normal physiological cellular function, but also one of the ‘effectors’ to feel the external stimuli [27]. Mitochondrial dysfunction induced by any factor results in the disorder of cell energy metabolism disorders, imbalance of the internal environment’s homeostasis of internal environment, and even cell death [28]. Mitochondria are the main site of ROS production, and the mitochondria-related components are preferred targets of ROS attacks. Moreover, due to the unique ‘geographical location’ of mitochondria, they are more vulnerable to oxidative damage than other organelles. This damage contributes to the abnormal internal structure and function of mitochondria, including effects on the respiratory chain, changes to the permeabilities of permeability on the inner and outer membranes, and altered expression of matrix proteins. These events trigger a series of cascade signals, thus initiating the development of intracellular mitochondrial autophagy and apoptosis [29]. Excessive ROS production in hepatocytes during oxidative stress not only induces the damage to nucleic acids, proteins, lipids, and other important components of cells but also activates multiple intracellular signaling pathways and promotes the development of inflammatory reactions, ultimately leading to cell apoptosis [30,31]. We found that the Docetaxel-induced cytotoxicity, mitochondrial dysfunction, and oxidative damage in LO-2 hepatocytes were significantly induced by Docetaxel and dramatically mitigated by Zafirlukast, accompanied by the amelioration of the apoptotic state in LO-2 hepatocytes, indicating that the Docetaxel-induced damages in hepatocytes were dramatically alleviated by Zafirlukast.

Inflammasomes play a critical role in the progression of innate immunity and inflammatory responses, thus, NLRP3 is widely investigated [32,33]. Following internal and external stimuli, the packaging and activation of the NLRP3 inflammasome is completed, inducing the transformation from inactivated pro-caspase-1 to activated caspase-1. Subsequently, the maturation of IL-1β and IL-18 is triggered by the activated caspase-1 and finally, the inflammatory cascade reaction is expanded [34,35]. In the present study, the activation of NLRP3 and the elevated release of IL-1β and IL-18 were observed in Docetaxel-treated hepatocytes. After the treatment with Zafirlukast, the activated NLRP3 and excessively released inflammatory factors were dramatically repressed, indicating that the protective effects of Zafirlukast on Docetaxel-stimulated hepatocytes might be associated with the inhibition of NLRP3-mediated inflammation.

SIRT1 is a NAD-induced class III histone deacetylase that is upregulated after RSV treatment. It is involved in various biological processes such as metabolism, oxidative stress, and inflammation [36]. Recent researches have shown that SIRT1 exerts a promising anti-inflammatory role by inhibiting the activation of the NLRP3 inflammatory complex [37,38]. Our data reveal that SIRT1 was upregulated in Docetaxel-stimulated hepatocytes after the treatment with Zafirlukast. Additionally, the impacts of Zafirlukast against Docetaxel-stimulated NLRP3 activation and apoptosis in LO-2 hepatocytes were greatly abolished by the silencing of SIRT1, indicating that Zafirlukast exerted its protective effects by upregulating SIRT1. In future work, the therapeutic effects of Zafirlukast on Docetaxel-induced liver injury will be further identified by establishing the animal model of DILI and checking the pathological changes after the administration of Zafirlukast.

## Conclusion

In the present study, we aimed to investigate the potential protective benefits of Zafirlukast in an *in vitro* DILI model. Our results uncovered that treatment with Zafirlukast protected hepatocytes against Docetaxel-induced mitochondrial dysfunction by inhibiting oxidative stress, the release of LDH, increase in Caspase-3 and Caspase-9 activity, as well as activation of the NLRP3 inflammasome. Importantly, we further demonstrated that the protective benefits of Zafirlukast in Docetaxel-challenged hepatocytes were mediated by SIRT1. Collectively, Zafirlukast ameliorated Docetaxel-induced cytotoxicity in hepatocytes by upregulating SIRT1.

## Data Availability

The data that support the findings of this study are available from the corresponding author upon reasonable request.
